# Pyrenoid-based CO_2_-concentrating mechanisms across diverse lineages: convergent features and their applications for engineering

**DOI:** 10.1093/plphys/kiag250

**Published:** 2026-04-29

**Authors:** Jessica H Pritchard, Sophie N R Young, Herbie Garland, Luke C M Mackinder, Alistair J McCormick

**Affiliations:** Institute of Molecular Plant Sciences, School of Biological Sciences, University of Edinburgh, Edinburgh, EH9 3BF, United Kingdom; Centre for Engineering Biology, University of Edinburgh, Edinburgh, EH9 3BF, United Kingdom; Institute of Molecular Plant Sciences, School of Biological Sciences, University of Edinburgh, Edinburgh, EH9 3BF, United Kingdom; Centre for Engineering Biology, University of Edinburgh, Edinburgh, EH9 3BF, United Kingdom; Centre for Novel Agriculture Products (CNAP), Department of Biology, University of York, Heslington, York, YO10 5DD, United Kingdom; Centre for Novel Agriculture Products (CNAP), Department of Biology, University of York, Heslington, York, YO10 5DD, United Kingdom; Institute of Molecular Plant Sciences, School of Biological Sciences, University of Edinburgh, Edinburgh, EH9 3BF, United Kingdom; Centre for Engineering Biology, University of Edinburgh, Edinburgh, EH9 3BF, United Kingdom

## Abstract

Engineering pyrenoid-based CO_2_-concentrating mechanisms (pCCMs) into crop plants is a promising strategy to boost photosynthetic performance, enhance yield potential, and improve resilience to future climates. Achieving this goal requires a deeper understanding of the molecular principles that enable pyrenoids to elevate CO_2_ around Rubisco. Although pyrenoids span a remarkable diversity of forms across algae and hornworts, they consistently exhibit 3 core features: a condensed Rubisco matrix, specialized membranes that deliver inorganic carbon, and a diffusion barrier that restricts CO_2_ leakage. Here, we review recent mechanistic advances from *Chlamydomonas reinhardtii* alongside emerging insights from other algae and hornworts that highlight both conserved strategies and lineage-specific innovations in Rubisco condensation, membrane-associated inorganic carbon channelling, and matrix encapsulation. Together, these findings refine our understanding of pCCM diversity and provide an increasingly robust blueprint for reconstructing pCCMs in vascular plant chloroplasts.

## Introduction

Ribulose bisphosphate carboxylase/oxygenase (Rubisco) is the primary carboxylation enzyme for assimilating CO_2_ into the biosphere. Rubisco captures CO_2_ by carboxylation of the CO_2_ acceptor molecule ribulose-1,5 bisphosphate (RuBP) to produce 2 molecules of 3-phosphoglycerate (3-PG). However, Rubisco can also catalyze a competing oxygenation reaction that produces a toxic by-product, 2-phosphoglycolate (2-PG), which must be removed by an energetically costly process called photorespiration. Apart from detoxifying 2-PG, photorespiration also plays a variety of important roles in carbon and nitrogen metabolism (Walker et al. 2024; Timm et al. 2025). However, the energetic costs of photorespiration limit the growth of many photosynthetic organisms, including most crops. Rubisco oxygenation rates and subsequent photorespiration also increase with increasing temperature, leading to further losses in photosynthetic carbon gain; this is a key concern considering the predicted rise in global temperature, as Rubisco performance will decline despite increasing CO_2_ levels (Walker et al. 2016; Zhu et al. 2023; Bernacchi et al. 2025). To reduce photorespiration, several organisms have evolved CO_2_ concentrating mechanisms (CCMs) to concentrate CO_2_ at the active sites of Rubisco, thus enhancing the efficiency of CO_2_ capture (Bräutigam and Gowik 2016; Raven et al. 2017). Much research has focused on engineering CCMs or photorespiratory bypasses into C3 plants as a key strategy to enhance crop yield potential and climate resilience (for reviews see Croce et al. 2024; Long et al. 2025).

One of the most abundant CCMs found in eukaryotic autotrophs is the pyrenoid-based CCM (pCCM). The pyrenoid is a chloroplastic phase-separated microcompartment that improves the efficiency of photosynthetic CO_2_ uptake by supplying Rubisco with concentrated CO_2_, and thus preferentially driving CO_2_ assimilation over photorespiration (He et al. 2023b). Pyrenoids are present across multiple lineages of algae and some nonvascular land plants (ie the hornworts) and appear to have evolved convergently, resulting in a wide range of structural diversity between species and clades (Villarreal et al. 2010; Meyer et al. 2017; Barrett et al. 2021; Long et al. 2024). Despite their varying architecture, pyrenoids typically have 3 common structural features: (i) a core matrix of condensed Rubisco; (ii) one or more matrix-traversing membranes or membrane invaginations that facilitate inorganic carbon (Ci; CO_2_ and HCO_3_^−^) delivery to Rubisco; and (iii) a barrier surrounding the condensate that is predicted to reduce leakage of CO_2_ (Fig. 1).

**Figure 1:**
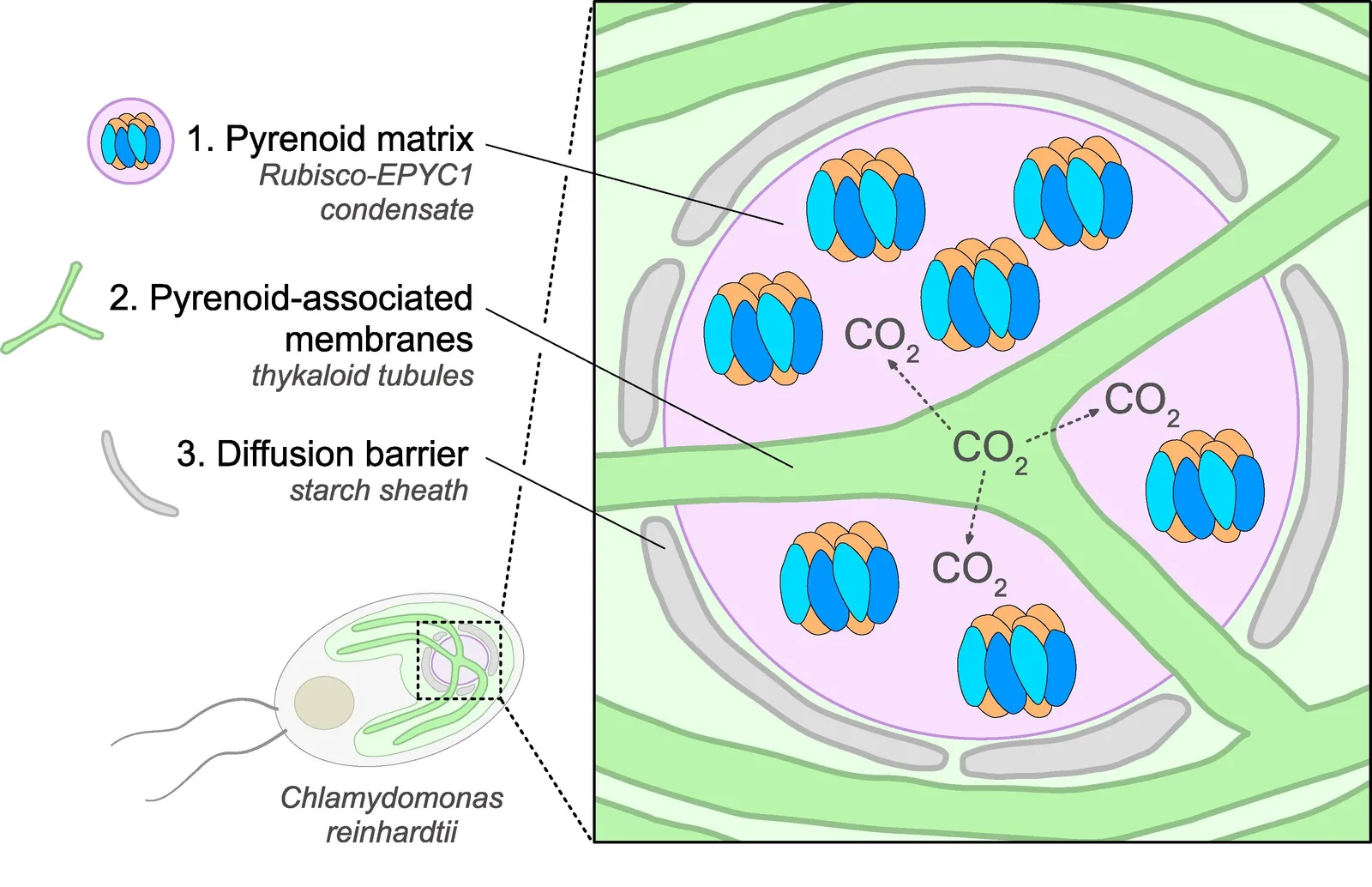
Pyrenoids are characterized by 3 common structural features. These are represented here in the pyrenoid of the model green alga *Chlamydomonas reinhardtii* as (1) a dense core matrix of Rubisco, (2) photosynthetic thylakoid membranes that traverse the Rubisco matrix, and (3) a surrounding sheath of starch plates. The matrix-traversing thylakoid membrane is thought to facilitate the supply of concentrated CO_2_ to the Rubisco matrix. The starch sheath is hypothesized to minimize leakage of CO_2_ out of the matrix and thus increase the operating efficiency of the pCCM (Fei et al. 2022).

Our molecular understanding of the pyrenoid and the associated components that supply Ci to the Rubisco matrix has largely been informed by work in the model green alga *Chlamydomonas reinhardtii* (Chlamydomonas hereafter). Recent work in Chlamydomonas and an array of diverse species, including diatoms, hornworts, and other algae, has now improved our fundamental knowledge of how pCCMs function and integrate with wider metabolism (Burcalot and Peltier 2023; You et al. 2025). The discovery and characterization of analogous and novel pCCM components in different species is also rapidly expanding the toolbox available to plant engineers aiming to build a functional pCCM in crop plants. Predictive models suggest that the introduction of a pCCM into C3 plant chloroplasts could increase the CO_2_ fixation potential of Rubisco by up to 3-fold (Fei et al. 2022), leading to high theoretical gains in crop yield potential (up to a 60% increase) (Long et al. 2015, 2019). Additional synergistic benefits include improvements in the efficiency of water use (WUE) and nitrogen use (NUE) per unit of biomass, and increased temperature resilience (up to a 10 °C increase in photosynthetic thermotolerance) (Bernacchi et al. 2025). Recently, an engineering road map for building a pCCM based on Chlamydomonas components has been proposed (Adler et al. 2022; Fei et al. 2022).

Here we discuss past and recent findings related to the Chlamydomonas pCCM and contextualize these with emerging insights from the discovery and characterization of pCCM components in other species. Structurally, we will consider these in 3 sections related to the common features highlighted above—Rubisco condensation, Ci delivery, and putative barriers to CO_2_ diffusion. Furthermore, we highlight how our growing molecular and mechanistic understanding of pCCM diversity might accelerate efforts to introduce functional pCCMs into crop plants.

## Condensation of Rubisco to form the pyrenoid matrix

### Current understanding of Rubisco condensation in Chlamydomonas

In Chlamydomonas the Rubisco matrix is assembled through weak multivalent interactions between the Rubisco small subunit (CrRbcS) and the linker protein Essential PYrenoid Component 1 (EPYC1) (Mackinder et al. 2016; Atkinson et al. 2019; He et al. 2020). When the pCCM is induced, EPYC1 is expressed at levels similar to Rubisco and condenses the stromal Rubisco pool into a single phase-separated condensate via the biophysical process of liquid–liquid phase separation (LLPS) (Hyman et al. 2014; Freeman Rosenzweig et al. 2017; Wunder et al. 2018). EPYC1 has 5 short helical Rubisco-binding motifs, or “stickers,” which interact with CrRbcS, interspersed by longer disordered “spacer” sequences (Fig. 2) (Ang et al. 2023). The interaction affinity between each EPYC1 sticker and CrRbcS is relatively low, reflected by a high dissociation rate (*K*_d_ = 3 mM for a 24-residue fragment; *K*_d_ = 414 μM for a 60-residue fragment) leading to transient binding and unbinding (He et al. 2020; Payne-Dwyer et al. 2024). However, each sticker may bind/unbind to a CrRbcS on a different Rubisco; this dynamic fluidity enables smaller Rubisco condensates to assemble into larger ones and allows internal mixing within the condensate (Freeman Rosenzweig et al. 2017; GrandPre et al. 2023; Catherall et al. 2025). Phase-separated condensates are also characterized by a surrounding dilute phase. In vitro and modelling work support that the dilute phase around the matrix consists of tightly bound EPYC1–Rubisco heterodimers (*K*_d_ = 30 nM) that coexist stably as equilibrium species (He et al. 2023b).

**Figure 2:**
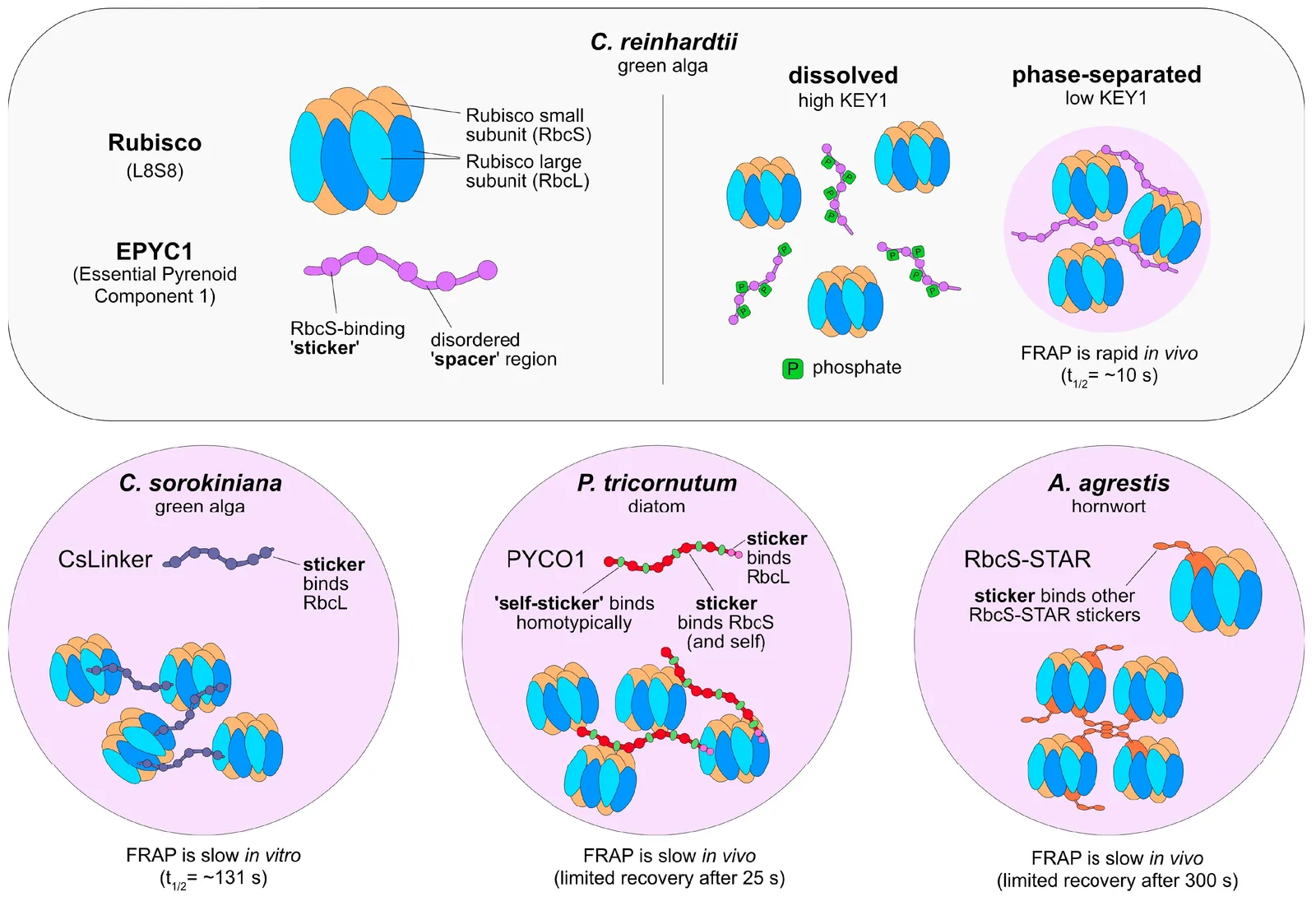
Rubisco condensation in the matrix is facilitated through a separate or an innate linker protein. Separate linker proteins have been identified in *Chlamydomonas reinhardtii* (EPYC1), *Chlorella sorokiniana* (CsLinker), and *Phaeodactylum tricornutum* (PYCO1). These proteins do not share a conserved sequence homology but do have common architectural features including regions of disorder (termed “spacers”) and repeating structured elements that bind to different regions of Rubisco (termed “stickers”). EPYC1 has 5 stickers, binds to the small subunit of Rubisco (RbcS) and is dynamically regulated by the KEY1 kinase (kinase of EPYC1), which phosphorylates EPYC1 and disrupts Rubisco binding resulting in dissolution of the condensate (He et al. 2026). CsLinker has 6 stickers and binds to the large subunit of Rubisco (RbcL) (Barrett et al. 2024). PYCO1 contains a complex array of stickers that can bind RbcS, RbcL, and itself (Oh et al. 2023). The hornwort *Anthoceros agrestis* expresses a specialized RbcS isoform with a C-terminal extension that functions as an innate Rubisco linker (RbcS-STAR). The STAR extension appears to condense Rubisco by interacting with STARs on adjacent Rubiscos to form tetrameric coiled-coils (Robison et al. 2026). Abbreviations: FRAP, fluorescence recovery after photobleaching; *t*_1/2_, half-maximal recovery time.

The factors that regulate the condensation, dissolution, and size of the Chlamydomonas Rubisco matrix during cell growth and division are still poorly understood. However, the kinase KEY1 was recently identified as a central regulatory component of these processes (He et al. 2026). Loss of KEY1 disrupts pyrenoid size and number, as well as normal matrix dissolution during cell division. He et al. (2026) showed that KEY1 localizes to the matrix and promotes dissolution by phosphorylating EPYC1 at multiple sites on and around the stickers, which reduces the binding affinity of the stickers for Rubisco. This results in partitioning of phosphorylated EPYC1 into the dilute phase, while dephosphorylated EPYC1 is enriched in the condensate (Fig. 2). The balance of these 2 phosphorylation states corresponds to a diel role for KEY1. During the light period KEY1 activity is low, maintaining the condensate whilst suppressing the formation of “ectopic” condensates. When cell division occurs at the shift from the light to the dark period, a rapid induction of KEY1 activity results in matrix dissolution. In newly formed daughter cells KEY1 activity is then reduced, allowing dephosphorylated EPYC1 to promote Rubisco condensation. A key next step will be to identify the component(s) that dephosphorylate EPYC1 and thus balance the regulatory activity and impact of KEY1 on the Rubisco matrix. Posttranslational control as a mechanism for linker regulation has yet to be reported in pyrenoids beyond Chlamydomonas. In the context of plant engineering, the discovery and characterization of KEY1 raises interesting questions regarding the extent of posttranslational regulation required for assembling and maintaining functional pyrenoids *in planta*.

### Uncovering the diversity of linker components that condense Rubisco in other species

Building on insights from Chlamydomonas, recent landmark studies in green algae, diatoms, and hornworts are rapidly expanding our understanding of the different mechanisms by which Rubisco is condensed into a matrix. Despite the critical role of EPYC1 in matrix condensation in Chlamydomonas, EPYC1 is not conserved outside closely related species. Thus, to identify functional analogues in other pyrenoid-containing species, Barrett et al. (2024) developed a computational pipeline to detect proteins characteristic of the structure of EPYC1, with repetitive disordered regions (ie spacers) followed by structured elements (ie stickers) spaced at a feasible scale for binding to different Rubisco complexes. The sequence homology-independent “Fast Linker Identification Pipeline for Pyrenoids” (FLIPPer) identified a putative linker in *Chlorella sorokiniana* that was later experimentally confirmed and named CsLinker.

CsLinker shares key architectural features with EPYC1, including α-helical domain stickers separated by intrinsically disordered spacer regions (Fig. 2). Subsequent structural analyses showed that CsLinker does not bind RbcS, like EPYC1, but instead interacts with the Rubisco large subunit (RbcL) (Barrett et al. 2024). In vitro, CsLinker appears to mediate Rubisco phase separation through a mechanism that mirrors EPYC1, although in vitro Fluorescence Recovery After Photobleaching (FRAP) experiments showed comparatively slower recovery (rates shown in Fig. 2). The estimated interaction affinity between a single CsLinker sticker and *C. sorokiniana* Rubisco was also substantially higher compared to EPYC1 and CrRbcS (*K*_d_ = 103 µM). Together, these data indicate reduced rates of mobility and increased internal cohesion within the *C. sorokiniana* Rubisco matrix compared to that observed in Chlamydomonas (Freeman Rosenzweig et al. 2017; Barrett et al. 2024). The biophysiological implications of these differences between pyrenoids are still being explored, while the implications, or preferences, for plant engineering efforts are yet to be established.

The RbcS interface to which EPYC1 binds is not conserved in plants, and EPYC1 cannot bind to native plant RbcSs to condense Rubisco (Atkinson et al. 2019). In contrast, CsLinker interacts with a generally well conserved RbcL interface (Barrett et al. 2024). This difference has important implications for plant engineering efforts. Current approaches using EPYC1 require co-expression of CrRbcS or a modified plant RbcS carrying the cognate CrRbcS helices to enable EPYC1–Rubisco interaction and “proto-pyrenoid” assembly in *Arabidopsis thaliana* (Arabidopsis hereafter) (Atkinson et al. 2019, 2020). CsLinker can phase-separate a broad range of plant and algal Rubisco in vitro, including those from ferns and the Solanaceae. Transient expression of CsLinker in *Nicotiana benthamiana* led to the formation of multiple Rubisco-associated puncta (Barrett et al. 2024), providing an initial proof-of-principle for its engineering potential. However, compared to CsRbcL, most angiosperm RbcLs (eg key C3 plant crops such as wheat, rice, soy) have a residue substitution within the CsLinker-binding interface that disrupts CsLinker-mediated Rubisco condensation. Further engineering of CsLinker to improve compatibility with non-Solanaceae Rubisco or additional screening of naturally occurring linkers that are compatible with non-Solanaceae Rubiscos are promising avenues to pursue. Going forward, key questions include whether CsLinker can assemble plant Rubisco into a single condensate (ie what ratio of CsLinker expression to Rubisco is required to achieve stable phase separation in vivo), and how CsLinker performs in stable transgenic systems.

Linker proteins have also been identified in several marine species with pyrenoids. In the model diatom *Phaeodactylum tricornutum*, Pyrenoid Component 1 (PYCO1) was identified as a matrix-associated protein that undergoes LLPS to form Rubisco condensates (Oh et al. 2023). PYCO1 is more complex than EPYC1 and CsLinker as it contains 5 and 6 repeats of 2 different “sticker” motifs, “KWSPRGGS” and “GTGYNP” respectively, separated by spacer regions (Fig. 2). The former sticker binds RbcS around the central solvent channel of Rubisco, while the latter allows for self-association of PYCO1 proteins. An additional motif near the C-terminus of PYCO1 was identified that binds RbcL, albeit relatively weakly compared to the repeated KWSPRGGS motif. PYCO1 displays slow recovery rates during FRAP experiments in vivo (>25 s), indicating low mobility within the Rubisco matrix. In contrast, in vitro experiments show that PYCO1 alone forms homotypic condensates with rapid FRAP recovery (ie a half-life recovery of 9 s). Further inclusion of Rubisco to form heterotypic condensates results in progressively slower FRAP recovery rates dependent on Rubisco concentrations (Fig. 2). This discrepancy suggests that PYCO1 may function as a scaffold protein within the matrix in vivo, where interactions with Rubisco and potentially other proteins within the matrix constrain PYCO1's mobility relative to its intrinsic biophysical properties. In terms of plant engineering, PYCO1 does not appear to be compatible with plant Rubiscos. In vitro analyses show that PYCO1 is highly specific for *P. tricornutum* Rubisco (form ID), and does not phase separate with Form II, Form 1B (Chlamydomonas or Arabidopsis), or form IC (*Rhodobacter sphaeroides*) Rubiscos (Oh et al. 2023).

A potential linker has been suggested in the chlorarachniophyte *Amorphochlora amoebiformis*, where a proteomic study of the pyrenoid identified AaPPAP28, a large protein that co-immunoprecipitates with Rubisco and localizes to the matrix (Moromizato et al. 2024). The central region of AaPPAP28 contains an unusual array of 90 short repeats consisting of 5 residues, separated by 2 to 23 residues (6 residues on average). It remains to be confirmed if the short repeats in AaPPAP28 are bona fide Rubisco stickers capable of phase separating Rubisco. If so, the overall architecture of AaPAPP28 would contrast to that of EPYC1 or CsLinker, which have 4 or 5 repeating stickers and average spacer intervals 52 and 48 residues, respectively (He et al. 2020; Barrett et al. 2024). Thus, AaPPAP28 could represent a novel linker architecture with repeating sequences restricted to the center of a larger protein.

Hornworts are the only identified group of land plants to assemble pyrenoids (Meyer et al. 2008; Li et al. 2020), and they provide another remarkable example of a divergent mechanism underlying Rubisco condensation. A putative analogue of EPYC1 was previously identified in the model hornwort *Anthoceros. agrestis*, but further analysis revealed that the candidate was unlikely to be a linker protein as it was not exclusively localized within the Rubisco matrix, was of low abundance, and evidence of interaction with Rubisco was inconclusive (Ruaud et al. 2025). More recent work has shown that LLPS of Rubisco in *A. agrestis* appears to be regulated not by a separate linker protein, but rather a specialized RbcS isoform that contains an extended C-terminal Sequestration-Associated Region (STAR), representing an exciting new class of linker (Fig. 2) (Robison et al. 2026).

RbcS-STAR is the second most abundant RbcS in *A. agrestis.* The STAR extension appears to enable direct interaction between Rubiscos by driving homotypic phase separation via coiled-coil interactions between 2 predicted α-helical domains. Robison et al. (2026) also demonstrated that RbcS-STAR could induce matrix formation when expressed in the closely related pyrenoid-lacking hornwort species *Anthoceros fusiformis* and in Arabidopsis. Furthermore, RbcS-STAR rescued the reduced growth phenotype of RbcS-deficient Arabidopsis mutants, indicating that it can assemble with RbcL to form functional Rubisco complexes *in planta* (Robison et al. 2026). FRAP experiments showed slow recovery rates for RbcS-STAR condensates, both in *A. agrestis* and Arabidopsis (Fig. 2). However, subsequent truncation of STAR in Arabidopsis to a single interacting motif resulted in significantly faster FRAP recovery, indicating that the STAR domain properties influence condensate dynamics. Thus, RbcS-STAR opens further interesting avenues for plant engineers to induce condensation of Rubisco and to modify the material properties of the Rubisco matrix.

Lastly, synthetic approaches are reinforcing the growing diversity of linker mechanisms. For example, fusion of the weakly dimerizing fluorescent protein superfolderGFP (sfGFP; Pédelacq et al. 2006) to RbcL in tobacco has been shown to generate pyrenoid-like condensates (Chen et al. 2025). Furthermore, tagging RbcL with sfGFP did not appear to impact Rubisco assembly or catalytic activity. Although the latter approach requires chloroplast transformation, which remains challenging in most plant species, these synthetic condensates highlight that stable Rubisco phase separation can be achieved through multiple biophysical routes, consistent with the convergent evolution of pyrenoid architectures across lineages (Barrett et al. 2021). De novo engineering approaches could adapt native plant proteins shown to drive condensation for many important cellular processes (for review see Emenecker et al. 2020) or heterologous systems; for example, components of the LAF-1 protein from *Saccharomyces cerevisiae* have been used to compartmentalize enzymes into cytosolic biomolecular condensates in *N. benthamiana* (Lindström Battle et al. 2026). Continued identification of new and diverse linkers will broaden the repertoire of molecular parts available for *in planta* pyrenoid engineering, increasing the feasibility of improving crop photosynthesis and potentially enabling species-specific tailoring of mechanisms and functional outcomes.

## CO_2_ delivery to the Rubisco matrix

Efficient delivery of Ci to the pyrenoid is essential for achieving elevated CO_2_ concentrations within the Rubisco matrix. In Chlamydomonas grown under ambient air conditions (approximately 0.04% CO_2_), data and modelling support that CO_2_ passively diffuses from outside the cell across the plasma membrane, aided by the CO_2_ channel CrLCI1 (Kono et al. 2020; Kono and Spalding 2020), and then across the chloroplast envelope into the chloroplast stroma where it is converted to HCO_3_^−^ by a carbonic anhydrase complex (CrLCIB and CrLCIC), driven by the high pH of the stroma (Wang and Spalding 2006; Yamano et al. 2010; Fei et al. 2022; Kasili et al. 2023) (Fig. 1). HCO_3_^−^ then passively diffuses via one or more selective bestrophin-like channels (CrBSTs) into the lumen of the thylakoid membranes. The thylakoid network has a high degree of interconnectivity across the chloroplast (Engel et al. 2015), allowing for direct diffusion of luminal HCO_3_^−^ into the specialized thylakoid tubules that traverse the Rubisco matrix, where another carbonic anhydrase (CrCAH3) converts HCO_3_^−^ back into concentrated CO_2_, now driven by the low pH of the lumen (Sinetova et al. 2012; Mukherjee et al. 2019). This concentrated CO_2_ then passively diffuses out of the traversing thylakoids to Rubisco in the matrix. The entire mechanism is powered by the pH differences between the compartments, which themselves are driven by the light reactions of photosynthesis (Raven 1997; Fei et al. 2022) (Fig. 3a). Under very low CO_2_ availability (<0.03% CO_2_), the pCCM in Chlamydomonas engages additional mechanisms for active uptake of extracellular HCO_3_^−^ through a series of channel proteins (Yamano et al. 2015) (for more detailed reviews see Wang et al. 2015; He et al. 2023a).

**Figure 3:**
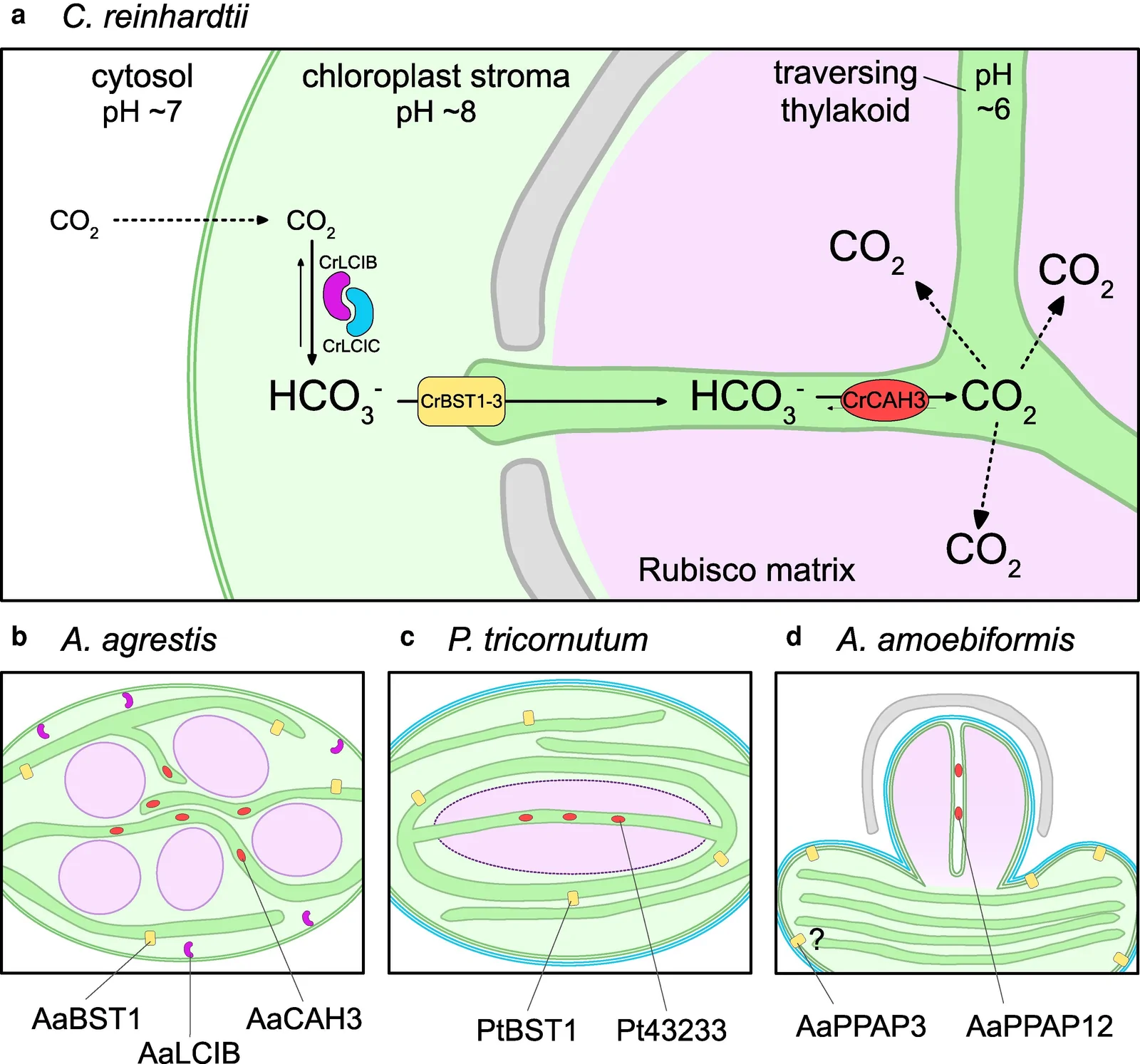
Ci delivery components appear common across diverse pyrenoids. The localization of carbonic anhydrases and bestrophin-like channels (BSTs) thought to be involved in Ci supply to the pyrenoid are shown for (a) *Chlamydomonas reinhardtii*, (b) *Anthoceros agrestis*, (c) *Phaeodactylum tricornutum*, and (d) *Amorphochlora amoebiformis*. In *C. reinhardtii*, CrLCIB is a stromal carbonic anhydrase that complexes with CrLCIC and is thought to capture CO_2_ as HCO_3_^−^ to supply Ci to the pyrenoid (Wang and Spalding 2014). CrLCIB/C is localized throughout the stroma at ambient CO_2_ and around the pyrenoid periphery at very low CO_2_ levels (Toyokawa et al. 2020). In *A. agrestis*, an orthologue of CrLCIB has been identified (AaLCIB) that is believed to have a similar role but is instead likely bound to the inner membrane of the chloroplast (Robison et al. 2025). Orthologues of CrBST1-3 have been identified in *A. agrestis* (AaBST1), *P. tricornutum* (PtBST1), and *A. amoebiformis* (AaPPAP30) and are similarly hypothesized to facilitate flux of HCO_3_^−^ into the membranes associated with the Rubisco matrix. The membrane localization of AaPPAP30 is not yet clear, but AaPPAP30 has been shown to associate with pyrenoid preparations. The traversing membranes are of thylakoid origin for *C. reinhardtii*, *A. agrestis*, and *P. tricornutum* and either traverse (*C. reinhardtii* and *P. tricornutum*) or closely appose (*A. agrestis*) the matrix. In *A. amoebiformis*, the pyrenoid is bisected by an invagination of the inner pair of plastid membranes into the matrix (ie the periplastidal compartment). Orthologues of CrCAH3 are localized to the thylakoid lumen of the pyrenoid-associated membranes for *A. agrestis* (AaCAH3), *P. tricornutum* (Pt43233), and in the periplastidal compartment between the inner and outer pair of plastid membranes in *A. amoebiformis* (AaPPAP12).

When comparing Ci delivery strategies across pyrenoid-containing lineages, past and recent studies indicate that 3 elements consistently emerge. First, the pyrenoid is associated with specialized membranes that traverse, partially penetrate, or closely appose the Rubisco matrix. These membranes are typically derived from thylakoids—though notable exceptions exist—and exhibit substantial variation in number, geometry, and organization (Meyer et al. 2017; Barrett et al. 2021; Moromizato et al. 2024). Second, bestrophin-like channels (BSTs) are frequently localized to or adjacent to the matrix-associated membranes, where they are presumed to mediate HCO_3_^−^ flux into the lumen (Mukherjee et al. 2019; Moromizato et al. 2024; Nigishi et al. 2024; Robison et al. 2025). Third, a carbonic anhydrase, analogous to CrCAH3, is positioned within the lumen of the matrix-associated membranes (Karlsson et al. 1998; Kikutani et al. 2016; Moromizato et al. 2024; Robison et al. 2025). Together, these conserved elements are beginning to provide a unifying framework for Ci delivery to the pyrenoid across diverse taxa (Fig. 3a, b). Below, we discuss these elements in more detail.

### Pyrenoid-associated membrane architecture

The membranes associated with the Rubisco matrix have long been proposed to facilitate CO_2_ delivery to the pyrenoid (Pronina and Borodin 1993). Their architecture varies markedly across lineages, indicating multiple independent solutions for generating a CO_2_-rich microenvironment within the Rubisco matrix (Barrett et al. 2021). In Chlamydomonas, thylakoid membranes extend into the matrix to form a complex network of pyrenoid tubules whose lumen is continuous with the thylakoid lumen (Engel et al. 2015; Hennacy et al. 2024; Elad et al. 2025). Furthermore, each tubule contains 2 to 8 smaller tubes called minitubules that appear to provide conduits from the interthylakoid stromal space to the matrix. Minitubules have not yet been observed in species other than Chlamydomonas, and their function and biogenesis are still poorly understood. However, they may facilitate the flux of Calvin cycle–related metabolites, such as RuBP and 3-PG, between the matrix and stromal compartments (Küken et al. 2018).

Tubular traversions also occur in *Chlorella luteoviridis* (Ikeda and Takeda 1995), but sheetlike matrix-traversing thylakoids appear more widespread across *Chlorella* spp., and pyrenoid-containing lineages in general. For example, the pyrenoids of *C. sorokiniana* and *C. saccharophila var. ellipsoidea* have one or many sheets of double-layered traversing thylakoids, respectively (Ikeda and Takeda 1995; Barrett et al. 2021). The Rubisco matrix in diatoms is typically traversed by one or two closely appressed thylakoid membranes, which may be continuous or possibly discontinuous with the rest of the thylakoid network (Bedoshvili et al. 2009). In the model diatoms *P. tricornutum* and *Thalassiosira pseudonana* these specialized membranes are termed the pyrenoid penetrating thylakoid (PPT) (Nam et al. 2024; Shimakawa et al. 2024). Multiple sheetlike traversions also occur in the hornwort *Notothylas breutelii* (Villarreal and Renner 2012). More unusual membrane architectures include the stacked thylakoid lamellae surrounding the segmented Rubisco matrices in the model hornwort *A. agrestis* (Robison et al. 2025), and the crystalline gyroid thylakoids of the green alga *Zygnema* sp. (Zhan et al. 2017). More rarely, matrix-traversing membranes may be of a non-thylakoid origin; for example, the Rubisco matrix in *A. amoebiformis* is bisected by the inner pair of plastid membranes (Moromizato et al. 2024).

The structural diversity of matrix-associated membranes may be indicative of an equally diverse set of molecular determinants. To date, only a few pyrenoid membrane-shaping factors have been identified and characterized. In Chlamydomonas, the scaffolding proteins STARCH GRANULES ABNORMAL (CrSAGA1) and MISSING THYLAKOIDS 1 (CrMITH1) have been shown to be required for the formation of matrix-traversing thylakoid tubules (Hennacy et al. 2024). Remarkably, co-expression of CrSAGA1 and CrMITH1 in Arabidopsis yielded sheetlike traversing thylakoids through the proto-pyrenoid matrix. However, the latter result also indicates that additional factors are likely required to reshape the membrane into tubules.

Recently, an affinity purification–based approach was developed to isolate and characterize the tubule proteome in Chlamydomonas (Franklin et al. 2026). This approach identified multiple proteins that were already known to localize to the tubule network, including CrBST4, CrMITH1, CrRBMP2, and CrCAH3 (Meyer et al. 2020; Adler et al. 2024; Hennacy et al. 2024). Additionally, several novel proteins and protein–protein interactions were revealed; for example, Pyrenoid Membrane Enriched 1 (CrPME1, Cre10.g452250) and Low-CO_2_-Induced 16 (CrLCI16, Cre02.g143550) were identified and shown to physically interact with each other. Furthermore, CrLCI16 was also found to interact with CrRBMP2, suggesting that these 3 proteins may form a novel membrane protein complex. Neither CrPME1 nor CrLCI16 was found to be essential for maintaining tubule morphology or for growth under low-CO_2_; thus, the components involved in tubule formation remain to be discovered. However, several proteins with homologues in other algal groups were identified, suggesting that there may be conserved mechanisms underlying matrix-traversing membrane formation.

### Functionalizing membranes for CO_2_ delivery to Rubisco

Insights from Chlamydomonas have provided a foundational model for understanding how the matrix-traversing thylakoid membranes deliver Ci to Rubisco. Recent studies are now revealing a striking convergence on the same core molecular strategy across multiple lineages. Despite substantial differences in membrane architecture, phylogenetic history, and chloroplast organization, growing evidence suggests that many species utilize BSTs as HCO_3_^−^ channels in conjunction with luminal carbonic anhydrases to generate a CO_2_-rich microenvironment around Rubisco.

In Chlamydomonas, 3 BSTs (CrBST1–3) appear to mediate HCO_3_^−^ flux into the lumina of matrix-traversing thylakoids (Mukherjee et al. 2019). Triple knockdowns of CrBST1–3 exhibit strong pCCM defects, but the partial redundancy among the isoforms suggests that a single BST may be sufficient to support an engineered system. Homologues of CrBSTs, with proposed similar functional roles, have now been identified across diverse pyrenoid-containing taxa, including diatoms *P. tricornutum* (PtBST1–4) and *T. pseudonana* (TpBST1–2), the chlorarachniophyte *A. amoebiformis* (AaPPAP30), and the hornwort *A. agrestis* (AaBST1) (Moromizato et al. 2024; Nigishi et al. 2024; Robison et al. 2025). Several of these isoforms are enriched in the thylakoid membranes at the pyrenoid periphery (eg AaBST1 [Robison et al. 2025], similar to CrBST1–3 [Mukherjee et al. 2019]), found throughout the stromal thylakoids (eg PtBST1 [Nigishi et al. 2024]) or are found throughout the pyrenoid traversing membranes (eg PtBST3 [Nigishi et al. 2024], TpBST1 and TpBST2 [Nam et al. 2026, 2024; Nigishi et al. 2024]). Knockout of PtBST1 resulted in a CCM defect with severely reduced Ci affinity in low CO_2_ grown cells (Nigishi et al. 2024). Collectively, it appears that bestrophin-like channels have been repeatedly recruited for the same physiological role of directing stromal HCO_3_^−^ into luminal compartments associated with the Rubisco matrix. Although additional functional characterization of these channels is still required, the recurrence of this strategy across major evolutionary lineages points to a convergent biochemical solution. This is promising from a plant engineering perspective as there is a range of functionally homologous candidates that may be employed to supply bicarbonate to an engineered pCCM in planta. If required, the activity of these candidates could then be fine-tuned to modulate bicarbonate flux to the pyrenoid (Guo et al. 2026).

A parallel theme of evolutionary convergence emerges for the presence of a luminal carbonic anhydrase in the matrix-associated membranes. Putative luminal-localized analogues of CrCAH3 have been identified in *P. tricornutum* (Pt43233), *T. pseudonana* (TpθCA2), *A. amoebiformis* (AaPPAP12), and *A. agrestis* (AaCAH3) (Kikutani et al. 2016; Nawaly et al. 2023; Moromizato et al. 2024; Robison et al. 2025). AaCAH3 is an α-carbonic anhydrase with sequence similarity to CrCAH3. In contrast, AaPPAP12, TpθCA2, and Pt43233 are classed as θ-carbonic anhydrases, one of the most recently discovered CA families, which possess a distinct active-site architecture yet exhibit catalytic properties broadly comparable to α-carbonic anhydrases (for review see DiMario et al. 2018). Modelling and functional studies underscore the importance of luminal CA activity. Modelling of the Chlamydomonas pCCM predicts that luminal carbonic anhydrase activity must be localized exclusively to the lumen of the membranes traversing the Rubisco matrix to avoid futile cycling of HCO_3_^−^ and CO_2_, which would otherwise reduce pCCM efficiency (Fei et al. 2022). Functional studies show that the CrCAH3-defective mutant *cia3* is unable to grow at ambient CO_2_ levels and accumulates elevated internal Ci pools, consistent with a bottleneck in CO_2_ release to the Rubisco matrix. (Moroney et al. 1986; Karlsson et al. 1998; Sinetova et al. 2012). Similarly, knockdown of the luminal θ-CA Pt43233 in *P. tricornutum* leads to reduced photosynthesis and impaired growth (Kikutani et al. 2016), reinforcing the view that luminal CA-mediated dehydration of HCO_3_^−^ is a conserved and essential step in sustaining pCCM function.

Further research into CrCAH3 function has shown that its activity and localization are also subject to regulatory control. Blanco-Rivero et al. (2012) provided evidence that localization of CrCAH3 to the pyrenoid is regulated by phosphorylation, but the underlying mechanism is still poorly understood. Under low pH, presumably when the pCCM is active, CrCAH3 adopts an unusual homodimeric structure in which N-terminal residues are exchanged between monomers (Benlloch et al. 2015). The functional relevance of this arrangement and the degree to which it is conserved across other luminal carbonic anhydrases remain to be resolved. Further biochemical characterization of CrCAH3 and CrCAH3 analogues represents a key area for future research, particularly in the context of engineering pCCMs into plants.

## Putative diffusion barriers around the Rubisco matrix

Pyrenoids across the algal and hornwort lineages employ a striking range of strategies to encapsulate the Rubisco matrix within a physical structural barrier, which is predicted to limit CO_2_ loss and as such is thought to be a key requirement for engineering an efficient pCCM in plants (Fei et al. 2022). These barriers can be broadly grouped into 3 types. First, many algal lineages employ polysaccharide-based barriers, such as a starch sheath or a polysaccharide cap (Barrett et al. 2021). Second, the secondary endosymbiont lineages derived from red algae, including diatoms, are characterized by protein-based barriers that also shape the matrix (Nam et al. 2024; Shimakawa et al. 2024). Lastly, some algae and the hornworts appear to utilize modified thylakoid architectures that are densely packed around or within the matrix (McKay and Gibbs 1990; Robison et al. 2025). These distinct solutions reflect the evolutionary diversity of pyrenoid organization and are discussed in more detail below.

### Polysaccharide-based diffusion barriers

A stromal starch sheath surrounding the Rubisco matrix is a characteristic feature of many green algal pyrenoids, including those of Chlamydomonas and *Chlorella* spp. (Fig. 4) (Ramazanov et al. 1994; Barrett et al. 2021). The sheath is typically composed of one or more curved starch plates, with gaps between the plates that permit thylakoid membranes to traverse the matrix and deliver Ci (Barrett et al. 2021; Hennacy et al. 2024). Although early studies in Chlamydomonas suggested that the starch sheath was not essential for pCCM function (Villarejo et al. 1996), more recent work has demonstrated a significant disruption to pCCM performance in starch-deficient mutants under low CO_2_ concentrations (Krishnan et al. 2015; Toyokawa et al. 2020). The prevailing model in Chlamydomonas suggests that the starch sheath serves as a diffusion barrier to reduce CO_2_ leakage away from the Rubisco matrix, which in turn increases the local concentration of CO_2_ and enhances the carboxylation efficiency of Rubisco (Fei et al. 2022).

**Figure 4:**
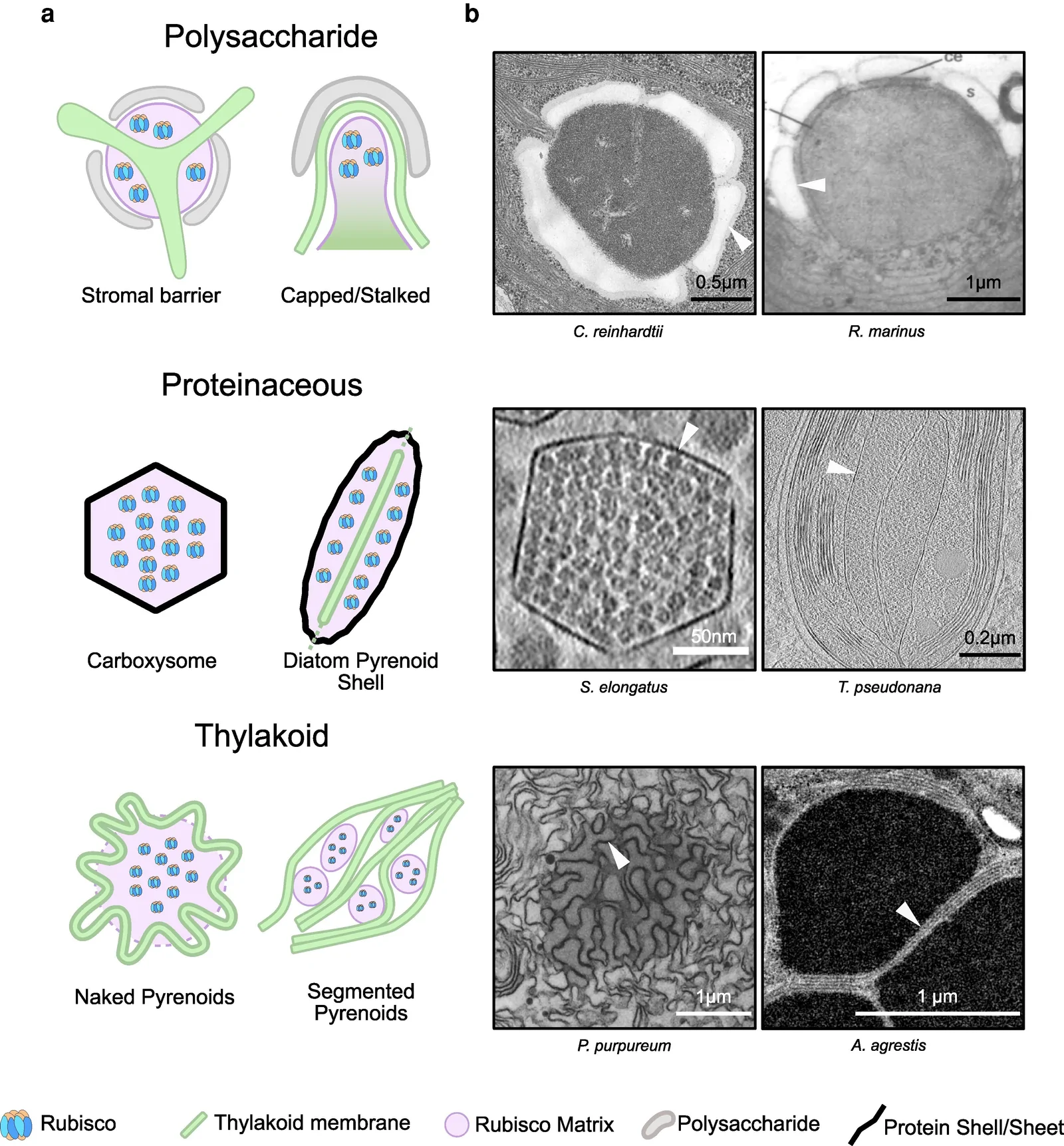
Putative diffusion barriers across algal and hornwort lineages. a) Examples of the diverse architecture of the barriers, which may be composed of polysaccharides (eg starch), proteins, or stacked thylakoid membranes. b) Example species are shown for each diffusion barrier class. White arrows have been added to indicate the potential diffusion barrier. References: *Chlamydomonas reinhardtii* (credit: Chun Sing Lau), *Rhodosorus marinus* (Ford 1984), *Synechococcus elongatus* PCC 7942 (Sun et al. 2025), *Thalassiosira pseudonana* (Shimakawa et al. 2024), *Porphyridium purpureum* (McKay and Gibbs 1990), *Anthoceros agrestis* (Robison et al. 2025).

A major challenge in studying the role of the starch sheath in Chlamydomonas pCCM function is that starchless mutants exhibit widespread structural and localization defects. For example, loss of the starch sheath leads to CrLCIB mislocalization, meaning it is difficult to distinguish whether the observed reduction in Ci affinity in the mutant is due to increased leakage of CO_2_ out of the pyrenoid in the absence of starch, or a functional defect in CrLCIB-driven CO_2_ capture (Toyokawa et al. 2020). Identifying mutants that lack a starch barrier or have increased starch permeability but retain proper localization of key CCM components may be crucial for disentangling the direct contribution of the starch sheath to CO_2_ retention.

To date, work in Chlamydomonas has highlighted several structural and starch metabolism–associated components that are known or predicted to play key roles in starch sheath synthesis and organization (Mackinder et al. 2017; Itakura et al. 2019; Meyer et al. 2020). CrSAGA1 (highlighted earlier for its additional role in the biogenesis of matrix-traversing thylakoids) and CrSAGA2 are large scaffolding proteins that localize to the starch–pyrenoid interface (Itakura et al. 2019; Meyer et al. 2020). Both contain an N-terminal CBM20 starch-binding domain and Rubisco-binding motifs, features that likely enable them to position the starch sheath adjacent to the Rubisco matrix. Notably, loss of CrSAGA1 has wide-ranging phenotypic impacts, including misshaped starch plates, multiple pyrenoids that lack traversing pyrenoid tubules, and a high CO_2_-requiring phenotype (Itakura et al. 2019). The expression of CrSAGA1 and CrSAGA2 in Arabidopsis containing a “proto-pyrenoid” led to the recruitment of starch granules to the “proto-pyrenoid” (Atkinson et al. 2024). However, additional factors are needed to shape the recruited starch granules into a sheath, which is presumably required to impart functionality.

More recently, Low CO_2_-Induced protein 9 (CrLCI9, recently renamed to Element for Pyrenoid Organization of Starch 1 [CrEPOS1]) has been shown to play a key role in starch sheath organization in Chlamydomonas (Mackinder et al. 2017; Adler et al. 2026). CrEPOS1 contains 2 CBM20 domains and localizes in a mesh-like structure around the periphery of starch plates, but is enriched at the plate interfaces. *epos1* mutants exhibit a reduced Ci affinity (ie an impaired pCCM) and are characterized by a disrupted sheath in which starch plates often overlap or misalign, indicating that CrEPOS1 is important for shaping of the starch sheath. When expressed in wild-type Arabidopsis, CrEPOS1 strongly associates with chloroplast starch granules, which suggests it may be a useful component to encourage starch shaping around a proto-pyrenoid in plants. Additional candidates in Chlamydomonas that warrant further investigation include Starch Branching Enzymes 1 and 4 (SBE1 and SBE4), which are part of the pyrenoid matrix interactome and may contribute to the unique curvature of starch plates by being spatially restrained to the matrix facing side of the starch sheath (Mackinder et al. 2017).

As an alternative to a central chloroplast location with a stromal starch sheath, many algae have pyrenoids that protrude, or “stalk,” out from the chloroplast towards the cytoplasm. The protruding chloroplast envelope(s) are surrounded by a cytoplasmic polysaccharide sheath, often referred to as a capping vesicle (Fig. 4). Capping vesicles are found in stalked pyrenoids across algal lineages, including red algae, brown algae, dinoflagellates, and chlorarachniophytes (Ford 1984; Hansen and Daugbjerg 2009; Hirakawa 2017; Moromizato et al. 2024). From these groups, only the chlorarachniophyte *A. amoebiformis* has been characterized at a molecular level, with proteomic analysis indicating 2 proteins, AaPPAP15 and AaPPAP21, that co-localize to the capping vesicle surrounding the pyrenoid (Moromizato et al. 2024). Stalked pyrenoids largely remain an understudied subgroup of pyrenoids with diverse structural variation from disparate algal clades. More information is needed on the assembly and function of capping vesicles.

### Protein diffusion barriers

Both prokaryotic and eukaryotic organisms have developed protein-based compartments that enclose Rubisco, which have been proposed to limit the diffusion of CO_2_ from its active site. Carboxysomes are well-characterized bacterial microcompartments that encapsulate Rubisco and carbonic anhydrase in a proteinaceous shell (Sun et al. 2024; Zhou et al. 2024). Molecular modelling suggests that the carboxysome shell may slow CO_2_ egress and O_2_ ingress, though experimental evidence remains limited (Liu 2022; Sarkar et al. 2024).

Recently, a novel protein lattice surrounding the pyrenoid was identified in the model diatoms *T. pseudonana* and *P. tricornutum* (Nam et al. 2024; Shimakawa et al. 2024). This has been termed the pyrenoid shell (PyShell), and is composed of PyShell proteins that are well conserved amongst red plastid secondary endosymbionts, including diatoms, pelagophytes, and haptophytes, suggesting a shared functional role (Nam et al. 2024). PyShell proteins assemble into a distinctive waffle lattice structure in diatoms that surrounds the Rubisco matrix and impart a lenticular shape to the pyrenoid (Fig. 4) (Shimakawa et al. 2024).

Unlike green algae, diatoms do not synthesize starch, so the absence of a discernible physical barrier complicated early efforts to understand their Ci flux (Hopkinson et al. 2011). Although it is not yet clear if the PyShell acts as a diffusion barrier for CO_2_, the discovery of PyShell proteins has helped to provide greater insight into carbon flux and the ultrastructure of diatom pyrenoids (Shimakawa et al. 2023, 2024; Nigishi et al. 2024; Shimakawa and Matsuda 2025; Himes and Kustka 2026).

Disrupting PyShell proteins leads to reduced growth and morphological changes in pyrenoid architecture. This includes increasing pyrenoid sphericity and loss of the pyrenoid penetrating thylakoid (PPT), suggesting that the PyShell is important for pyrenoid organization and function in diatoms. Perturbation of the PPT is particularly detrimental as this leads to mislocalization of the luminal θ-CA, a carbonic anhydrase required for diatom photosynthesis even under elevated CO_2_ (Nawaly et al. 2023; Shimakawa et al. 2023, 2024).

Diatom PyShells also highlight how structural pyrenoid barriers may function as organizational hubs for other CCM components, as seen from the many shell-interacting proteins (Nam et al. 2024). As with starch-sheath mutants in Chlamydomonas, the multifaceted defects observed in PyShell mutants complicate efforts to disentangle the roles of the shell as a diffusion barrier and/or a spatial organizer. Nonetheless, the self-assembling nature of PyShell proteins makes them an attractive alternative candidate for engineering diffusion barriers around proto-pyrenoids in plants.

### Thylakoid diffusion barriers

The diversity of pyrenoid architectures across algae suggests that multiple diffusion-barrier mechanisms have evolved independently, but many of these remain poorly characterized. Several lineages appear to rely on thylakoid-based barriers that differ significantly from the polysaccharide- or protein-based systems described above. Here we discuss 2 examples.

Red algae (Rhodophyta) are a large but understudied group of photosynthetic eukaryotes that display a wide variety of pyrenoid morphologies. In *Porphyridium purpureum* (previously *Porphyridium cruentum*), the pyrenoid is “naked” and lacks any obvious biophysical boundary with the surrounding chloroplast stroma (Fig. 4) (Brody and Vatter 1959). Instead, a dense and highly curved thylakoid network traverses the Rubisco matrix. Such networks may act as diffusion barriers analogous to the stacked thylakoids predicted to restrict CO_2_ movement in Chlamydomonas models (Fei et al. 2022). To date, molecular details on Rhodophyte pyrenoids, like that in *P. purpureum*, remain sparse, and more work is needed to elucidate components involved in their organization and function.

Finally, similar to Rhodophyta, pyrenoid morphologies in the hornworts are highly diverse. However, several are characterized by multiple Rubisco matrices segmented by thylakoids (Vaughn et al. 1992). Pyrenoid adjacent stromal starch granules can be observed in the chloroplasts of some, but not all, pyrenoid-containing hornworts; however, these granules are unlikely to form an effective diffusion barrier because of the large gaps between granules. The model pyrenoid-containing species *A. agrestis* has no visible starch sheath in the chloroplast and is characterized by a segmented pyrenoid surrounded by stacks of thylakoid membranes (Vaughn et al. 1992; Villarreal et al. 2010; Robison et al. 2025). As with *P. purpureum*, our understanding of how the thylakoid membranes could contribute to CO_2_ retention to improve pCCM performance in hornworts is still limited to analogous in silico modelling studies (Fei et al. 2022).

## Conclusion

A growing body of evidence from diverse pyrenoid-bearing lineages reveals that despite striking architectural variation, pyrenoids are built around a recurring combination of Rubisco condensation, Ci-delivering membranes, and diffusion barriers. Comparative studies now highlight a shared biochemical strategy in which BST-type HCO_3_^−^ channels and luminal carbonic anhydrases work together to generate a CO_2_-rich microenvironment around Rubisco.

For engineering efforts, this convergence is encouraging as it implies that a limited set of components, appropriately targeted and regulated, may be sufficient to recreate a functional pCCM in a vascular plant chloroplast. Continued exploration of pyrenoid diversity will therefore be crucial for accelerating efforts to engineer pCCMs into crops to improve photosynthetic performance and resilience.

Advances Box

- Several novel Rubisco linker proteins and CO2-concentrating mechanism components have been identified and characterised in diverse pyrenoidcontaining species.
- The cross-species reactivity of the Rubisco linker protein CsLinker can drive condensation of some native plant Rubiscos.
- A novel “innate” Rubisco linker protein has been discovered in hornworts.
- The kinase KEY1 is a central regulatory component of pyrenoid condensation and dissolution in Chlamydomonas.

Outstanding Questions

- Can linker proteins from species with broad RbcL compatibility (eg CsLinker) assemble plant Rubiscos into a single matrix *in vivo*?
- Which features of linker proteins or domains (eg sticker number, spacer length and flexibility, coiled-coil dynamics) determine condensate material properties, and how might these influence pCCM performance?
- How do regulatory mechanisms (eg phosphorylation by KEY1) that control Rubisco condensation vary across diverse lineages?
- What molecular factors determine whether traversing membranes form as tubules or sheets?
- How do minitubules form, what is their function, and do analogous structures exist in species outside Chlamydomonas?
- Are BST analogues from diverse taxa functionally equivalent, and which isoforms are most suitable for plant engineering?
- How is the localization of CrCAH3 regulated?
- Do PyShell protein lattices act as CO2 diffusion barriers?
- Why are pyrenoids only found in hornworts and not in vascular plants?
- Which C3 crops stand to benefit most from an engineered pCCM, and what factors determine compatibility?

## Data Availability

No new data were generated or analysed in support of this research.
